# Cellular dynamics of regeneration reveals role of two distinct Pax7 stem cell populations in larval zebrafish muscle repair

**DOI:** 10.1242/dmm.022251

**Published:** 2016-06-01

**Authors:** Tapan G. Pipalia, Jana Koth, Shukolpa D. Roy, Christina L. Hammond, Koichi Kawakami, Simon M. Hughes

**Affiliations:** 1 Randall Division of Cell and Molecular Biophysics, Guy's Campus, King's College London, London SE1 1UL, UK; 2 Weatherall Institute of Molecular Medicine, John Radcliffe Hospital, Oxford University, Oxford OX3 9DS, UK; 3 Division of Molecular and Developmental Biology, National Institute of Genetics, andDepartment of Genetics, SOKENDAI (The Graduate University for Advanced Studies), Mishima, Shizuoka 411-8540, Japan

**Keywords:** Myotome, Myogenesis, Myogenin, Myoblast heterogeneity, Fusion, Somite, Satellite cell, Injury

## Abstract

Heterogeneity of stem cells or their niches is likely to influence tissue regeneration. Here we reveal stem/precursor cell diversity during wound repair in larval zebrafish somitic body muscle using time-lapse 3D confocal microscopy on reporter lines. Skeletal muscle with incision wounds rapidly regenerates both slow and fast muscle fibre types. A swift immune response is followed by an increase in cells at the wound site, many of which express the muscle stem cell marker Pax7. Pax7^+^ cells proliferate and then undergo terminal differentiation involving Myogenin accumulation and subsequent loss of Pax7 followed by elongation and fusion to repair fast muscle fibres. Analysis of *pax7a* and *pax7b* transgenic reporter fish reveals that cells expressing each of the duplicated *pax7* genes are distinctly localised in uninjured larvae. Cells marked by *pax7a* only or by both *pax7a* and *pax7b* enter the wound rapidly and contribute to muscle wound repair, but each behaves differently. Low numbers of *pax7a*-only cells form nascent fibres. Time-lapse microscopy revealed that the more numerous *pax7b*-marked cells frequently fuse to pre-existing fibres, contributing more strongly than *pax7a*-only cells to repair of damaged fibres. *pax7b*-marked cells are more often present in rows of aligned cells that are observed to fuse into a single fibre, but more rarely contribute to nascent regenerated fibres. Ablation of a substantial portion of nitroreductase-expressing *pax7b* cells with metronidazole prior to wounding triggered rapid *pax7a-*only cell accumulation, but this neither inhibited nor augmented *pax7a*-only cell-derived myogenesis and thus altered the cellular repair dynamics during wound healing. Moreover, *pax7a*-only cells did not regenerate *pax7b* cells, suggesting a lineage distinction. We propose a modified founder cell and fusion-competent cell model in which *pax7a*-only cells initiate fibre formation and *pax7b* cells contribute to fibre growth. This newly discovered cellular complexity in muscle wound repair raises the possibility that distinct populations of myogenic cells contribute differentially to repair in other vertebrates.

## Abstract (precis)

**Summary:** Tracking muscle precursor cells during wound repair with confocal time-lapse microscopy reveals that two myoblast populations fuse and contribute differentially to regeneration, one initiating and the other growing fibres.

## INTRODUCTION

Efficient wound repair is key to vertebrate survival and thus under strong evolutionary selection. In skeletal muscle, wounds, surgery, degenerative diseases, or even the high forces generated during running downhill, trigger damage that is repaired from satellite cells, resident muscle stem cells that lie beneath the basal lamina of healthy muscle fibres (Mauro, 1961). During repair, satellite cells activate to form proliferative myoblasts. Some lineage descendants of these satellite cell-derived myoblasts regenerate fibres by cell cycle exit, terminal differentiation and fusion, but other satellite cell progeny self-renew, returning to quiescence. Molecular mechanisms involved in satellite cell-dependent muscle fibre repair are increasingly understood, mainly through studies in rodents and in tissue culture cells (reviewed in Ciciliot and Schiaffino, 2010; Yin et al., 2013). For example, recent studies have highlighted the importance of the transcription factor Pax7 as a marker of satellite cells and a key regulator of the repair process itself (Gunther et al., 2013; Seale et al., 2000; von Maltzahn et al., 2013). It remains unclear, however, whether all satellite cells are equal or whether distinct classes of muscle precursor cell (MPC) contribute to distinct aspects of muscle regeneration.

The difficulty of imaging the muscle repair process in the live animal has hampered efforts to analyse muscle stem cell contributions to repair. With this in mind, a number of groups have turned to the zebrafish, in which optical clarity permits lineage tracing and monitoring of individual identified cells *in vivo* over long periods. Like other teleosts, zebrafish efficiently repair muscle wounds (Knappe et al., 2015; Li et al., 2013; Otten et al., 2012; Rodrigues et al., 2012; Rowlerson et al., 1997; Seger et al., 2011) and accumulation of Pax7-expressing cells in wounds has been described (Knappe et al., 2015; Seger et al., 2011). Zebrafish models of several muscle-degenerative diseases have been developed (Bassett et al., 2003; Gupta et al., 2011, 2012; Ruparelia et al., 2012; Sztal et al., 2012; Wallace et al., 2011) and their regeneration analysed (Seger et al., 2011). Moreover, satellite cells marked by Pax7 have been reported in a variety of teleost species, including zebrafish (Hollway et al., 2007; Zhang and Anderson, 2014; reviewed in Siegel et al., 2013).

Developmentally, satellite cells originate from the dermomyotome of the somite, a transient embryonic structure that is also marked by expression of Pax7, and its close paralogue Pax3 (Gros et al., 2005; Kassar-Duchossoy et al., 2005; Relaix et al., 2005). The teleost equivalent of dermomyotome, an external cell layer of Pax3- and Pax7-expressing cells on the lateral somite surface, exists in zebrafish and contributes to muscle growth (Devoto et al., 2006; Groves et al., 2005; Hammond et al., 2007; Hollway et al., 2007; Stellabotte et al., 2007; Waterman, 1969). Dermomyotomal cells reside on the somite surface, where they divide and are thought to contribute cells that participate in later muscle growth (Hammond et al., 2007). Such cells have also been shown to contribute to repair of wounds in larval muscle (Knappe et al., 2015; Seger et al., 2011).

Here we employ the larval zebrafish as an *in vivo* model to characterise the heterogeneity of satellite cells in skeletal muscle wound repair. We demonstrate that in the wounded somite several distinct fibre types begin to regenerate within two days. Time-lapse confocal imaging shows that muscle repair is a dynamic process in which several waves of cells successively invade the wounded tissue. During this process Pax7-expressing cells show a burst of proliferation, followed by accumulation of the muscle-specific transcription factor Myogenin and differentiation to repair and regenerate fibres. Numerous Pax7-expressing mononucleate cells persist within the regenerated somite. Cells expressing either *pax7a* or *pax7b* gene reporters each contribute to repair, but behave differently. Cells expressing *pax7a* only and those expressing *pax7a* and *pax7b* accumulate, differentiate and fuse distinctly within wounds. The results lead us to hypothesise that *pax7a*-only cells preferentially initiate nascent fibres, whereas *pax7b*-expressing cells more commonly fuse to repair and grow fibres.

## RESULTS

### Time course of muscle repair in larval zebrafish

**Fig. 1.**
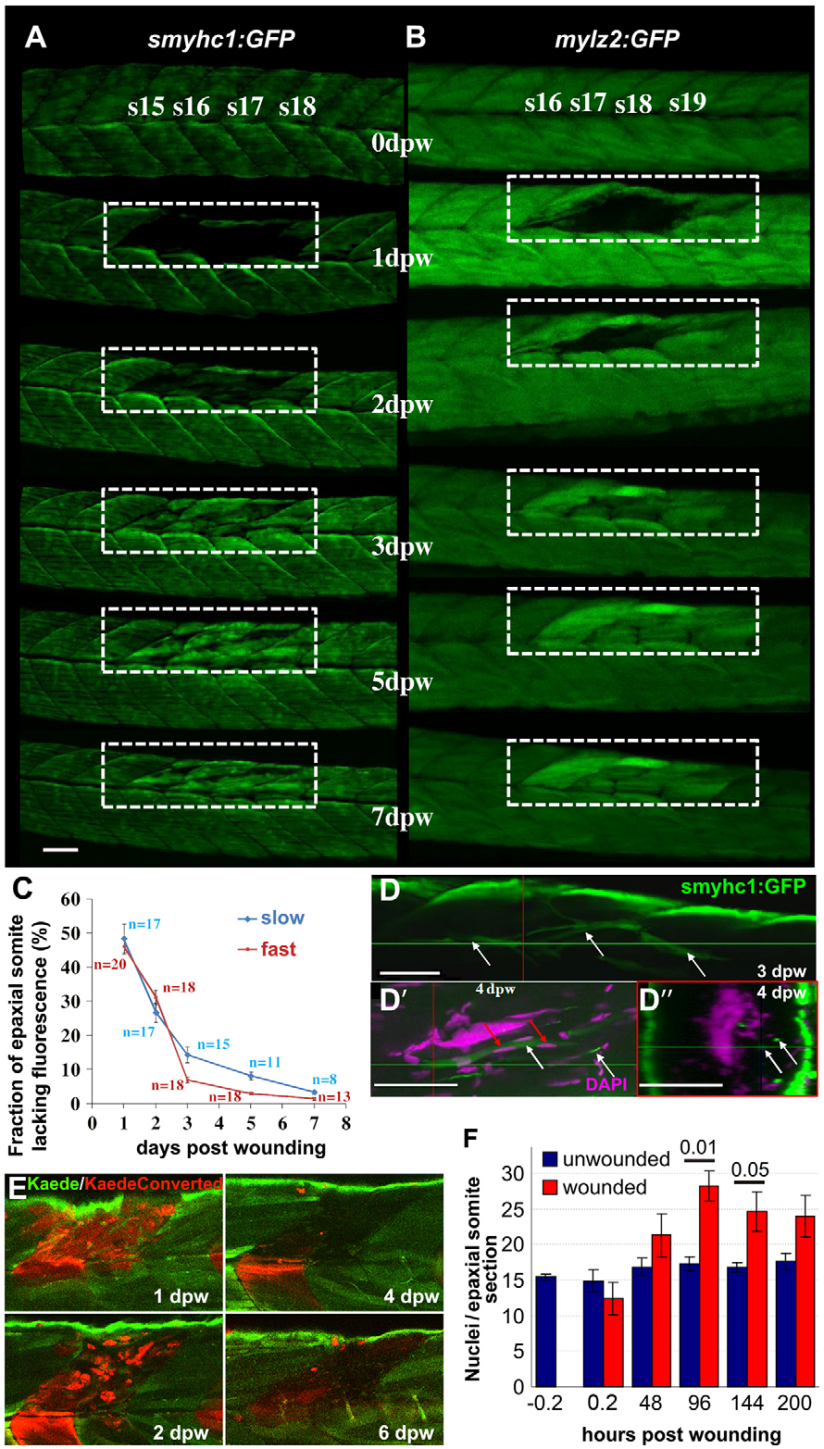
**Time-course of muscle wound repair.** (A,B) Large needle incision wounds (boxed regions) in the indicated somites of zebrafish 3.5 dpf larvae carrying transgenes expressed in slow (A; *smyhc1:gfp*) or (B; *mylz2:gfp*) fast fibres were repeatedly imaged in the same live fish by confocal fluorescence microscopy over 7 dpw. Larvae are shown anterior to left, dorsal up. Note the brighter fluorescence of newly synthesised unbleached GFP in regenerated region. s15-s19, somite 15 to somite 19. (C) Rate of recovery (mean±s.e.m.) of GFP fluorescence in epaxial somite of slow *smyhc1:gfp* and fast *mylz2:gfp* muscle of *n* larvae. (D-D″) *smyhc1:gfp* larvae showing slow fibres (white arrows) in deep somite, viewed from dorsal (D; 3 dpw) and lateral (D′) and corresponding transversal (D″) views at 4 dpw. The red and green crosshairs indicate planes, red arrows indicate elongated fibre-associated nuclei*.* (E) To investigate the source of new fibres, two adjacent somites in embryos injected with Kaede RNA were photoconverted from green to red at 2.5 dpf, then wounded in the epaxial domain and followed for 6 dpw. Representative confocal slices in lateral view show loss of KaedeRed without replacement by KaedeGreen. (F) Loss and gain of nuclei (mean±s.e.m.) in epaxial somites of *Tg(h2afva:H2AFVA-GFP)^kca66^* larvae wounded at 3.5 dpf and imaged until 12 dpf (ANOVA, *n*=4). Scale bars: 50 µm.

Zebrafish larvae expressing GFP in specific muscle fibre types were wounded by unilateral needle insertion into the epaxial somite. *Tg(9.7kb smyhc1:gfp)^i104^*, in which the *slow myosin heavy chain 1* enhancer drives GFP labelling of ∼20 mononucleate superficial slow muscle fibres in each somite (Elworthy et al., 2008), and *Tg(-2.2mylz2:gfp)^i135^*, which labels underlying multinucleate fast fibres (von Hofsten et al., 2008), were used to analyse fibre loss and repair in individual fish over time (Fig. 1A-C). Upon lesion, GFP fluorescence rapidly diminished in the disrupted fibres. At 1 day post-wounding (1 dpw), significant loss of labelled fibres was observed in one to three somites in each transgenic line. Contralateral and adjacent somites seemed unaffected (Fig. 1A,B and data not shown). By 2 dpw, small smyhc1:GFP and mylz2:GFP fibres were observed spanning the wound region. Reappearance of GFP in both slow fibre monolayer and underlying fast muscle was significant by 3 dpw (Fig. 1A-C). Although fibres generally re-integrated correctly into the original somite structure, some misplaced slow fibres were observed deep in the wound site (Fig. 1D-D″). Kaede photoconversion-based cell tracking revealed that the vast majority of labelled cells at the injury site were lost and replaced by weakly fluorescent cells between 2-4 dpw (Fig. 1E), thereby showing that Kaede tracing was not suitable to determine the source of regenerated muscle fibres (Fig. 1E). Analysis of wounded larvae stained with phalloidin and Hoechst 33342 confirmed the loss of structural components of muscle (Fig. S1A). Nuclei within the lesion were rapidly lost and then re-accumulated at the wound site from 2 dpw onwards (Fig. S1B; Fig. 1F). Thus, damage to somitic muscle fibres is rapidly repaired, consistent with previous reports (Rodrigues et al., 2012; Seger et al., 2011). These findings show that the cell biology of muscle wound repair is open to time-lapse analysis in zebrafish embryos.

**Fig. 2.**
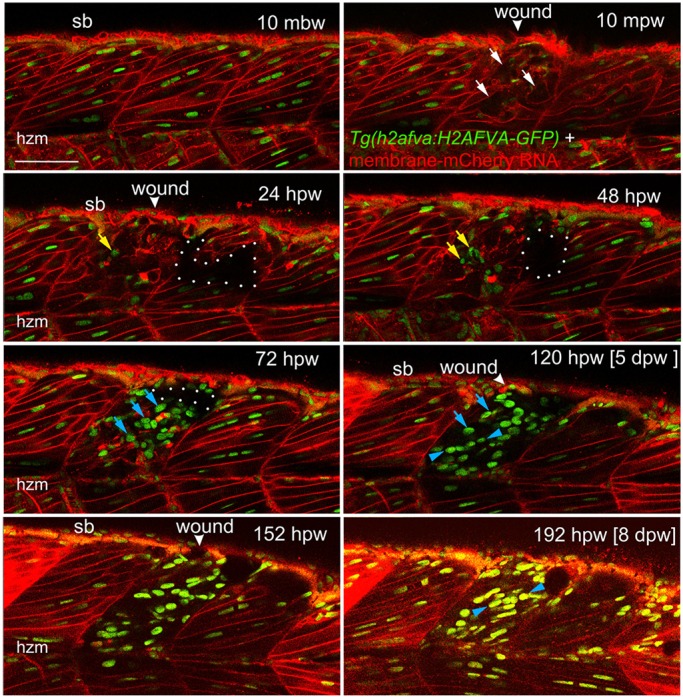
**Muscle fibre regeneration in confocal time-lapse microscopy.** Larvae from the *Tg(h2afva:H2AFVA-GFP)^kca66^* line injected with membrane-mCherry RNA were wounded in epaxial somite 17 at 3.5 dpf and imaged by 3D confocal time-lapse microscopy for 200 hpw at 22°C. Parasagittal views are single optical slices at indicated time points from the full time series (see Movie 1). Disruption of fibres is evident immediately after wounding (white arrows). Scan shadow cast by a melanophore migrating near the wound is outlined (white dots). After loss of elongated fibre nuclei, cells with small round nuclei accumulate in wound (yellow arrows). Photobleaching resulting from scanning is evident at later times, but abundant large nuclei are located in wounds after 48 hpw (blue arrows). By 5 dpw, numerous rows of bright aligned nuclei are apparent (blue arrowheads). mbw, mpw, hpw and dpw: minutes before, or minutes, hours or days post-wounding; hzm, horizontal myoseptum; sb, somite border. Scale bar: 50 µm.

To understand muscle cell behaviour during regeneration in more detail, 3.5 days post-fertilisation (dpf) larvae labelled in nuclei with histone-GFP and plasma membrane with mCherry were analysed by time-lapse confocal microscopy for 8.5 days (*n*=5) (Fig. 2; Movie 1). Again, we observed a clear disruption of muscle structure immediately after wounding and fluorescence decreased (Fig. 2). By 24 hours post-wounding (hpw), the shape of nuclei within the wound was more heterogeneous than in equivalent unwounded regions. Cells with small round GFP^+^ nuclei, morphologically unlike those in unwounded fibres, arose in the wound region. Between 24-72 hpw, some of these cells had intense membrane mCherry signal, moved rapidly, and were probably leukocytes (Fig. 2; Movie 1 and see below). Less motile rounded GFP^+^ nuclei persisted beyond 72 hpw, becoming more numerous over subsequent days (Movie 1), with some showing mitotic profiles. Membrane mCherry signal diminished in the wound after 48 hpw, paralleling degradation of fibre components (Fig. 1E). From 3-8 dpw, some nuclei within the wound had higher GFP signal than their neighbours in adjacent unwounded imaged somites, potentially reflecting synthesis of new histone-GFP during cell proliferation (Fig. 2). By 120 hpw (5 dpw), rows of aligned nuclei were observed in the wound (Fig. 2). Despite some persistent disorganisation of the wounded somite, aligned elongated nuclei characteristic of mature fibres accumulated after 5 dpw. Like Kaede, membrane mCherry remained low in the regenerating wound, presumably because regenerating fibres could not re-synthesise the markers due to a lack of residual injected RNA. Taken together, the time course of the events during larval zebrafish muscle regeneration parallels that of adult mammal muscle regeneration.

### Rapid epidermal closure and leukocyte infiltration to muscle wounds

Avoidance of bacterial infection is a key element of the response to injury. We observed that epidermal lesions closed rapidly, within 1 h in a purse-string fashion in the case of single somite-width needle lesions (Fig. S2A-C). Moreover, as in the case of simple epidermal wounds or muscle degeneration (Richardson et al., 2013; Walters et al., 2009), leukocytes (marked by *lyz* and *mpx* transgenes and therefore probable neutrophils) infiltrated the wound within 2 hpw (Fig. S2D,E). Brightly mCherry-fluorescent cells, putative phagocytes, entered the wound within 20 min (Fig. S2F). These appear to be invading leukocytes that transiently occupied the wounded somite, constituting a small fraction of the ∼160 total nuclei in an epaxial somite at 48 hpw, and then leave during the 36-60 hpw period (Fig. 1E; Fig. S2E,F). Thus, most nuclei in regenerating somites are not leukocytes.

### Nuclear loss and recovery during muscle regeneration

Despite the invasion of leukocytes, total nuclear number transiently decreased in wounded epaxial somites shortly after injury and remained reduced at 24 hpw, presumably resulting from the degradation of damaged tissue (Fig. 1F; Fig. S1). Thereafter, average nuclear number recovered, reaching 125% of control or adjacent uninjured somites (Fig. 1F). In somites with smaller wounds, nuclear number did not increase, whereas larger wounds generally led to a significant excess of nuclei compared with adjacent unwounded somites (Fig. 1F). We conclude that proliferation and/or migration of cells into the myotome contribute to the regeneration of somitic muscle.

### Wounding triggers proliferation and differentiation of Pax7 cells within the somite

**Fig. 3.**
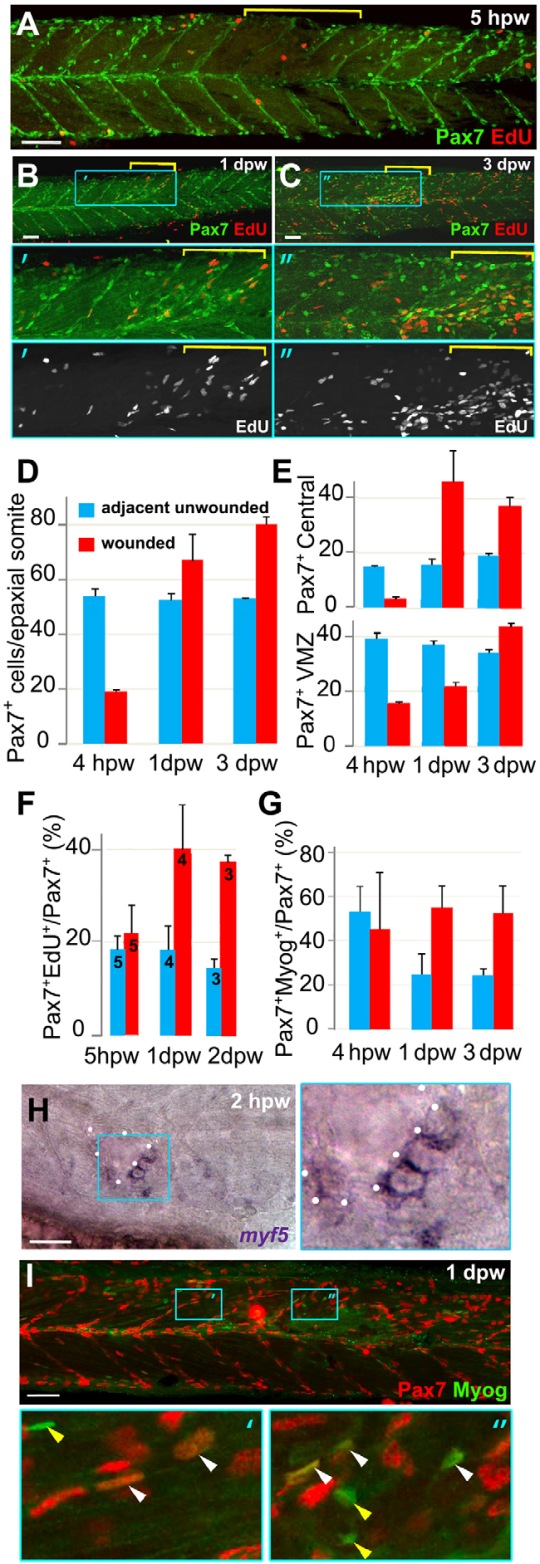
**Rapid recovery of Pax7-expressing cells in wounded somites through proliferation and relocation enhances differentiation in central myotome.** Wild-type zebrafish larvae wounded at 3 dpf in epaxial somites 16-18 (yellow brackets) were analysed at the indicated times post-wounding by confocal immunodetection of Pax7 with EdU (A-F) or Myogenin (G,I) or *in situ* mRNA hybridisation for *myf5* (H), shown in lateral view, anterior to left, dorsal to top. Blue boxes are magnified. (A-C) Diminished numbers of Pax7^+^ cells after wounding (A) are rapidly replaced (B) and show increased proliferation (B,C). (D-G). Pax7^+^ cells were counted in 2-4 wounded and 2-4 adjacent unwounded somite regions per larva and averaged to yield a value for each animal. VMZ, vertical myoseptum. Mean±s.e.m values from four larvae (D,E,G) or the number indicated (F). Statistical analysis is shown in Fig. S3A. (H) *myf5* mRNA adjacent to a hypaxial wound (outlined by dots). Note the lack of *myf5* mRNA in unwounded somites at this stage. (I) Pax7^+^Myog^+^ nuclei (white arrowheads) generally have lower Myog signal than Pax7^−^Myog^+^ cells (yellow arrowheads). Scale bars: 50 µm.

In addition to muscle fibres, at the time of wounding, somites contained mononucleate cells, many of which are marked by the muscle stem/precursor cell marker Pax7 (Hammond et al., 2007; Hollway et al., 2007; Minchin et al., 2013; Stellabotte et al., 2007; Windner et al., 2012). These Pax7^+^ cells are originally distributed on the lateral myotome surface and concentrate at the dorsal and ventral edges of the somite and the horizontal and vertical myosepta (HZM and VMZ; Windner et al., 2012). Subsequently, small numbers of Pax7^+^ cells arise in the deep central myotome (Minchin et al., 2013) (Fig. 3A). Upon making a large wound, the number of Pax7^+^ cells was rapidly reduced and then recovered by 1 dpw (Fig. 3A,B,D; Fig. S3A). At 1-3 dpw, an increased proportion of Pax7^+^ cells were in S-phase, as assayed by EdU pulse labelling (Fig. 3B,C,F). To demonstrate that proliferative cells contribute to new muscle fibres in wounds, larvae marked with membrane-targeted GFP were continuously exposed to EdU from 3 hpw to 3 dpw. At regenerating wounds, large numbers of nuclei were observed, most of which were EdU^+^ and new fibres at the wound contained multiple EdU^+^ nuclei (Fig. S4). Fibres in adjacent unwounded somites contained few EdU-labelled and many unlabelled nuclei (Fig. S4), indicative of a low rate of MPC fusion to muscle fibres during growth. Most nuclei in regenerating muscle wounds had undergone S-phase after wounding. Thus, proliferation of Pax7^+^ cells contributes to recovery in somite cell numbers.

The location of Pax7^+^ cells changed after wounding. Whereas in control or adjacent unwounded somites, Pax7^+^ cells remained predominantly at somite borders and HMZ, numerous Pax7^+^ cells arose in the central myotome after wounding (Fig. 3B,C,E; Fig. S3A), consistent with the observations of Seger et al. (2011), Furthermore, in wounded somites, Pax7^+^ cells at the VMZ recovered more slowly than those in the central myotome, even though Pax7^+^ cells at the VMZ rapidly entered S-phase after wounding (Fig. 3E; Fig. S3D,E). Analysis of cells at the VMZ of wounded somites in time-lapse series of histone-GFP fish injected with membrane-mCherry RNA revealed unusual cells with a single small nucleus and bright polar mCherry aggregates, particularly in deep somite regions (Fig. S5). These cells could not be tracked in this wounding time series, prompting us to develop live markers for Pax7^+^ cells (see below). A parsimonious hypothesis to explain these observations is that Pax7^+^ cells on vertical somite borders become activated to proliferate and migrate into the central region of damaged somites.

To examine the differentiation state of Pax7^+^ cells, we analysed myogenic transcription factor expression in wounds. Within 2 hpw, *myf5* mRNA, a marker of myogenic progression, was detected in cells adjacent to wounds (Fig. 3H). Unlike *myf5*, which marks myoblasts, Myogenin protein (Myog) is a marker of terminal MPC differentiation (Hinits et al., 2011; Weinberg et al., 1996). Immediately after wounding, fewer Myog^+^ cells were present in wounded somites, paralleling the loss of all cell types examined (data not shown). However, at both 1 and 3 dpw, more Myog^+^ cells were present in the central region of damaged somites than in undamaged adjacent somites (Fig. 3G,I). Moreover, the fraction of Pax7^+^ cells co-expressing Myog was increased compared with adjacent unwounded somites, as was the number of cells expressing Myog alone (Fig. 3G; Fig. S3B,C). Similar co-expression of Pax7 and Myog has been reported previously (Day et al., 2009; Devoto et al., 2006). These findings suggest that, despite the increased proliferation of the Pax7^+^ cell population, Pax7^+^ cells in wounds were more frequently undergoing terminal differentiation than those in unwounded regions.

### Pax7-expressing cells contribute to muscle regeneration

**Fig. 4.**
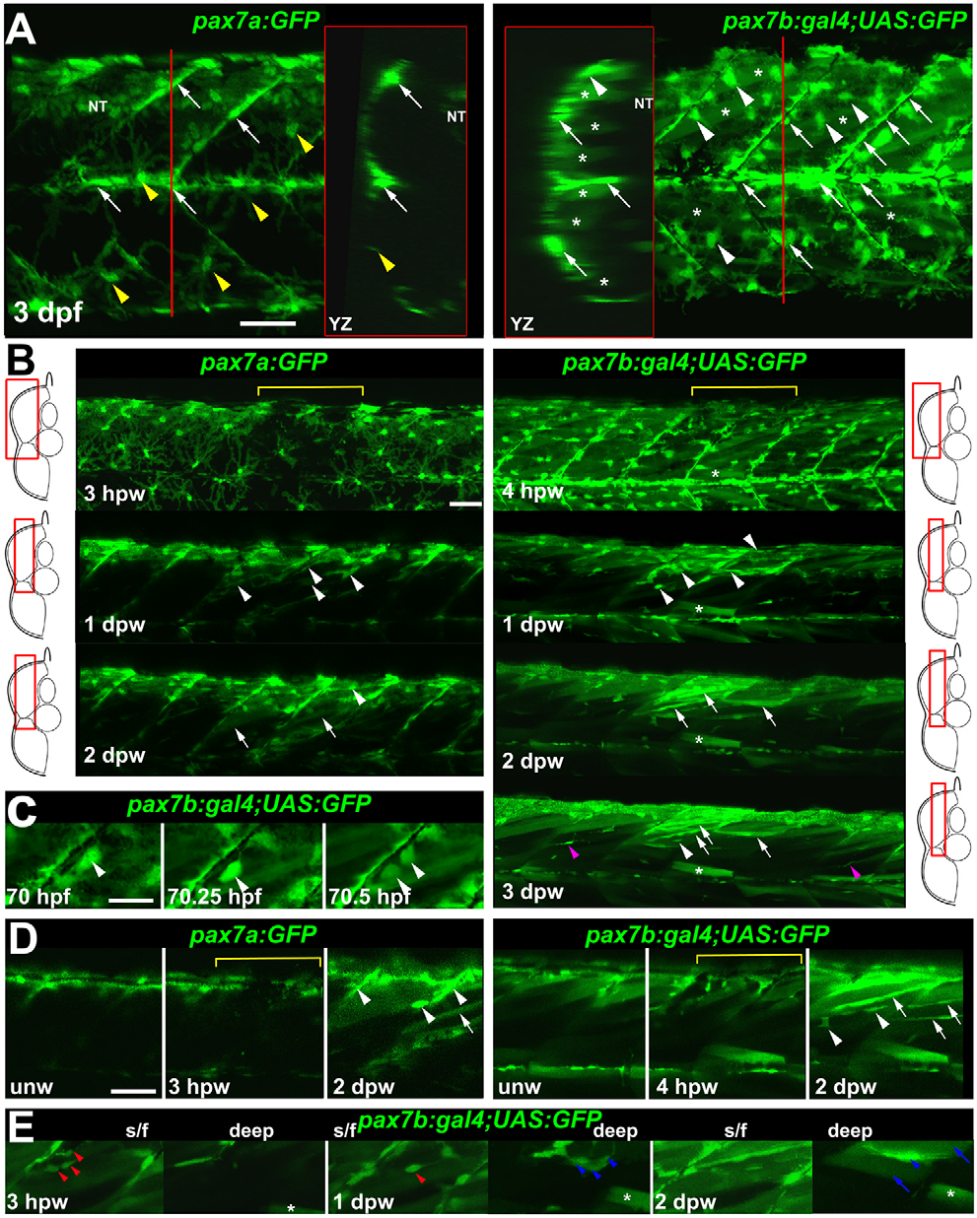
***pax7a*- and *pax7b*-reporter transgenes express distinctly during wound repair.** (A) Confocal maximum intensity projection of stacks of whole somites in lateral view, showing the distribution of GFP^+^ cells at 3 dpf in unwounded *pax7a:GFP* and *pax7b:gal4;UAS:GFP* fish. Note xanthophores (yellow arrowheads), dermomyotomal cells (white arrowheads), VMZ and HZM cells (white arrows), and labelled fibres (asterisks). Transverse *YZ* slices were taken at the red line. NT, neural tube. (B) Live confocal time lapse imaging of short stacks taken from the volumes indicated in the adjacent transverse schematics, shown in lateral view, anterior to left, dorsal up. Note loss of signal at 3-4 hpw in regions wounded at 3 dpf (yellow brackets), and recovery of GFP^+^ cells (arrowheads) and muscle fibres (arrows) over the ensuing days. Asterisk indicates persistence of a deep fast fibre marked by *pax7b*-reporter prior to wounding. Unwounded somites also accumulate small numbers of marked mononucleate cells (magenta arrowheads). (C) Time series confocal slices showing *pax7b*-reporter^+^ cell division (arrowheads) prior to wounding. (D) Magnified confocal slices showing wounding (yellow brackets) and repair. Note the stronger fibre labelling (arrows) with *pax7b*-reporter than with *pax7a:GFP*, relative to mononucleate cells (arrowheads). (E) Time series confocal slices showing superficial (s/f, left panels) and deep (right panels) *pax7b*-reporter^+^ cell appearance in the wound region followed by fusion. Disappearance of small bright GFP^+^ cells amongst the superficial fibres (red arrowheads) correlated with appearance of small bright GFP^+^ cells in deep regions (blue arrowheads, centre). Loss of some small deep cells then correlated with appearance of weakly GFP-labelled fibres (blue arrows; rightmost panel). Asterisks indicate a deep fast fibre marked by *pax7b*-reporter prior to wounding. Scale bars: 50 µm.

**Fig. 5.**
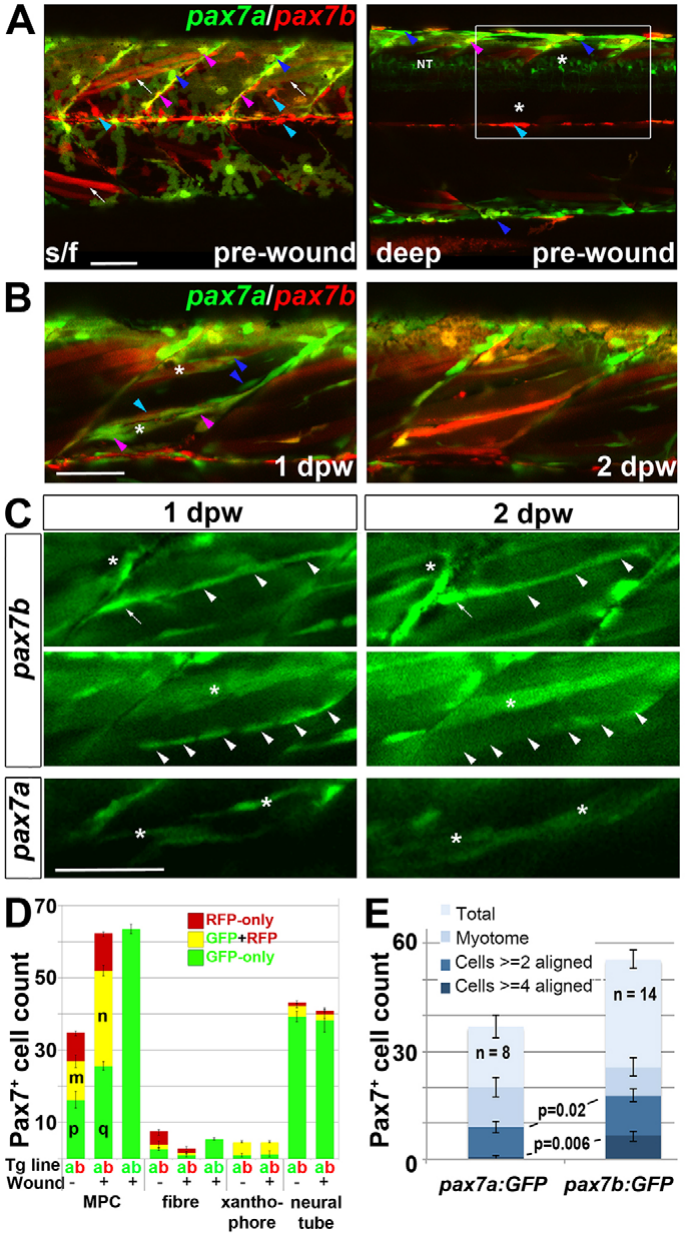
**Fusion of *pax7a*- and *pax7b*-reporter cells during wound repair.** (A-C) Lateral confocal maximum intensity projection stacks of pre-wounded (A) and wounded (B,C) yolk extension somites of *pax7a:GFP;pax7b:gal4;UAS:RFP* (A,B) or single *pax7a/b:GFP* (C) larvae, anterior to left, dorsal to top. Scale bars: 50 µm. (A) At 3 dpf, *pax7b:RFP* fibres (white arrows) and presumptive mononucleate cells (cyan arrowheads) are present superficially (s/f) within the somite and differ from *pax7a:GFP* cells (blue arrowheads). Dual-labelled somite cells (magenta arrowheads) concentrate on VMZ. Note the lack of Pax7 cells in the deep myotome at this stage. The *pax7b*-reporter labelled cells strongly in somites, and also weakly in dorsal neural tube (NT). (B) Short stack of epaxial wounded region shown by white box in A with two small wounds (asterisks). At 1 dpw, *pax7a:GFP;pax7b:RFP* cells elongate in wound. By 2 dpw, time-lapse reveals several nascent fibres marked strongly by RFP and weakly by GFP. See Fig. S9 for separate monochrome images. (C) Time-lapse of *pax7b:gal4;UAS:GFP* reporter marks aligned cells (arrowheads) that form fibres (top) or disappear (centre). *pax7a:GFP* cells are frequently aligned with fibres, but more rarely assemble in rows. *pax7a:GFP* cells occasionally matured into nascent fibres (bottom). Asterisks mark the same cells at each time point. Note the stronger mononucleate cells and more abundant fibre labelling by the *pax7b:GFP* reporter, compared with the *pax7b:RFP* reporter in panel B. Arrow indicates a separate cell. (D) Counts of numbers (mean±s.e.m.) of red, green and dual-labelled cells in a single epaxial somite (or corresponding length of neural tube) by cell type in larvae transgenic (Tg) for *pax7a:GFP* (a, green), *pax7b:GFP* (b, green) or *pax7b:RFP* (b, red) as indicated by the Tg line letter code and colour. Larvae with (+) or without (−) a wound made at 3 dpf were analysed 1 dpw, at 4 dpf. Note the increase in labelled MPCs, decrease in fibres and constant number of xanthophores and neurons in wounded somites at 1 dpw. Letter groups (m,n,p,q) indicate difference at *P*<0.05 (*t*-test, *n*=3). (E) At 1 dpw, despite a similar fraction of total cells in myotome, there were more *pax7b:GFP* reporter cells in rows of two (≥2) or four (≥4) or more aligned cells, compared with *pax7a:GFP* cells. Mean±s.e.m., *P*-values show Mann–Whitney test of differences in proportions of total cells.

To verify that Pax7^+^ cells contribute to muscle regeneration, we employed fish labelled with a *pax7a:GFP* BAC transgene (Mahalwar et al., 2014; S. Alsheimer, PhD thesis p. 249, Universität Tübingen, 2012). Prior to wounding, and in control and adjacent unwounded somites, the reporter labelled cells on the somite borders, as well as xanthophores and cells in the dorsal neural tube. To examine the response of MPCs specifically, *pax7a:GFP* was bred onto a *pfeffer* mutant background that substantially reduces xanthophore number (compare Fig. 4A with Fig. 5A) (Odenthal et al., 1996). In large wounds, most *pax7a:GFP* signal was lost at the wound site, consistent with ablation of many MPCs (Fig. S6A). *pax7a:GFP* cells re-accumulate at 1 dpw, divide and migrate, gradually invading the wound and contributing to fibres near the wound edge by 2 dpw (Fig. S6). Correlating with the extent of wound and time course of *pax7a:GFP* cell invasion, repair rate varied. However, by 6 dpw muscle seemed regenerated and some *pax7:GFP* cells remained undifferentiated after recovery (Figs S6, S7). Thus, *pax7a*-expressing MPCs participate in muscle wound repair.

### Pax7a- and Pax7b-expressing cells behave differently

We observed a mismatch between transgenic *pax7a:GFP* labelling and Pax7 immunoreactivity. In large wounds, Pax7 protein was detected in more cells within the central wound by 1 dpw, than expressed *pax7a:GFP* (Fig. 3B,D, Fig. 4B; Fig. S6A, Fig. S7). Zebrafish have duplicated Pax7 paralogues, *pax7a* and *pax7b*, which are both expressed in somitic cells (Minchin and Hughes, 2008). We therefore examined a *pax7b* splicing trap reporter line (*gSAIzGFFD164A*;5x*UAS:EGFP*; Fig. S8) for the response of cells expressing *pax7b* to muscle wounding. At 3 dpf in this line (hereafter referred to as *pax7b:GFP*), strong GFP was observed in cells in or near the myosepta, both VMZ and HZM. GFP was also detected in numerous superficial fast muscle fibres (Fig. 4A) (Minchin and Hughes, 2008). This markedly contrasted with *pax7a:GFP*, which rarely marked fibres in unwounded conditions (Fig. 4A). Conversely, *pax7a:GFP* strongly marked xanthophores, but *pax7b:GFP* was weaker in these cells. Instead, *pax7b:GFP* was observed in numerous dermomyotome cells overlying the slow fibres (Fig. 4A). Whereas *pax7a:GFP* cells predominated at the dorsal edge of the myotome, *pax7b:GFP* cells were concentrated at the HZM (Fig. 4A,B). Thus, *pax7a:GFP* and *pax7b:GFP* were differentially regulated, prompting the question of their expression in wounds.

Time-lapse 3D confocal microscopy of wounds revealed differences between *pax7a:GFP* and *pax7b:GFP* MPCs. Upon wounding, both pax7a:GFP^+^ and pax7b:GFP^+^ cells were reduced in numbers, but already by 1 dpw re-accumulated in the wound region (Fig. 4B). Like pax7a:GFP^+^ cells, pax7b:GFP^+^ cells were observed to proliferate (Fig. 4C). Short time-lapse analyses showed that both pax7a:GFP^+^ and pax7b:GFP^+^ cells often migrate into the myotome from somite borders (data not shown). Fibre labelling in wounds was consistently more pronounced in *pax7b:GFP* than in *pax7a:GFP* fish. Whereas about half of new pax7b:GFP^+^ fibres were brighter than surrounding marked mononucleate cells, this was not the case for pax7a:GFP^+^ fibres (Fig. 4B,D; Fig. S6C, Fig. S7). In wounds, pax7b:GFP^+^ cells were observed to fuse to large, presumably pre-existing, fibres, as well as to contribute to thin nascent fibres (Fig. 4E). Fusion to pre-existing fibres was very rarely observed for pax7a:GFP^+^ cells (data not shown). Most strikingly, *pax7a:GFP* rarely marked large regenerating fibres within wounds, and then only weakly, suggesting that the GFP in nascent fibres is not stable enough to persist in larger maturing fibres (Fig. 4D). In contrast, *pax7b:GFP* readily marks maturing fibres in wounds, both superficial and deep, and these were brighter than with *pax7a:GFP*, suggesting that *pax7b:GFP*-expressing cells contribute to the growth of regenerated or damaged fibres (Fig. 4D,E). To summarise, cells marked by either *pax7a* or *pax7b* each contribute to larval muscle wound repair.

To understand the different behaviour of *pax7a*- and *pax7b*-marked cells better, *pax7a:GFP;gSAIzGFFD164A;5xUAS:RFP* (*pax7a:GFP;pax7b:RFP*) larvae were examined. To reduce the complexity of wound repair dynamics, smaller focal wounds were made by fine needle insertion into a local region of a single somite. Such wounds had a more uniform repair time course and facilitated imaging and were therefore used in all subsequent experiments. MPCs contained either pax7a:GFP, pax7b:RFP or both (Fig. 5A,D). Most *pax7a:GFP*-only cells were located at the dorsal myotome edge or VMZ, whereas most *pax7b:RFP*-only cells were located at the HZM (Fig. S9A). Upon wounding, both *pax7a:GFP* and *pax7b:RFP* cells accumulated in the wound at 1 dpw, with most cells expressing both markers. Cells expressing only *pax7a:GFP* were also observed within wounds (Fig. 5B; Fig. S9B). GFP in *pax7a:GFP*-only cells was in general brighter than in dual-labelled cells. Counts revealed that *pax7a:GFP*-only and *pax7a:GFP;pax7b:RFP* dual-labelled MPCs accumulated in wounded somites at 1 dpw (Fig. 5D). *pax7b:RFP*-only cells did not increase in numbers and were rare within wounds. Thus, the presence or absence of *pax7b:RFP* distinguished two MPC populations within wounds.

In contrast to 1 dpw, by 2 dpw, each gene marked regenerated fibres within the wound region (Fig. 5B). Almost all newly formed fibres in *pax7a:GFP;pax7b:RFP* larvae at 2 dpw had detectable RFP and GFP, although their GFP was generally weak (Fig. 5B; Fig. S9B, see also Movies 2, 3). Moreover, clusters of large fibres acquired labelling in the wound region, but not elsewhere, and tended to look red. Such newly marked fibres augmented the pre-existing red fibres. (Fig. 5B; Movie 2). These data show that *pax7a:GFP*-only cells behave differently from *pax7b:RFP* cells (most of which also express *pax7a:GFP*), with the latter contributing more strongly to fibres in wounds.

To compare the contribution of each cell population to fibre repair, the response of mononucleate MPCs in identified wounded fish was examined at 1 dpw and again at 2 dpw. At 1 dpw, *pax7b:GFP* cells were numerous and frequently observed in rows of up to seven cells within the wound region aligned with fibres, but subsequently disappeared, being replaced by marked fibres. The orientation of such rows subtly changed with depth within the somite, matching the orientation of fast fibres in unwounded somites (Fig. 5C and data not shown). In contrast, although the bright *pax7a:GFP*-only MPCs were motile, no groups had more than four aligned MPCs (Fig. 5C,E). Moreover, such MPCs within wounds at 1 dpw did not form large numbers of *pax7a:GFP*-only fibres at 2 dpw (Fig. 5B,C). Thus, dual-labelled cells aligned and seemed to fuse more often than *pax7a:GFP*-only cells.

**Fig. 6.**
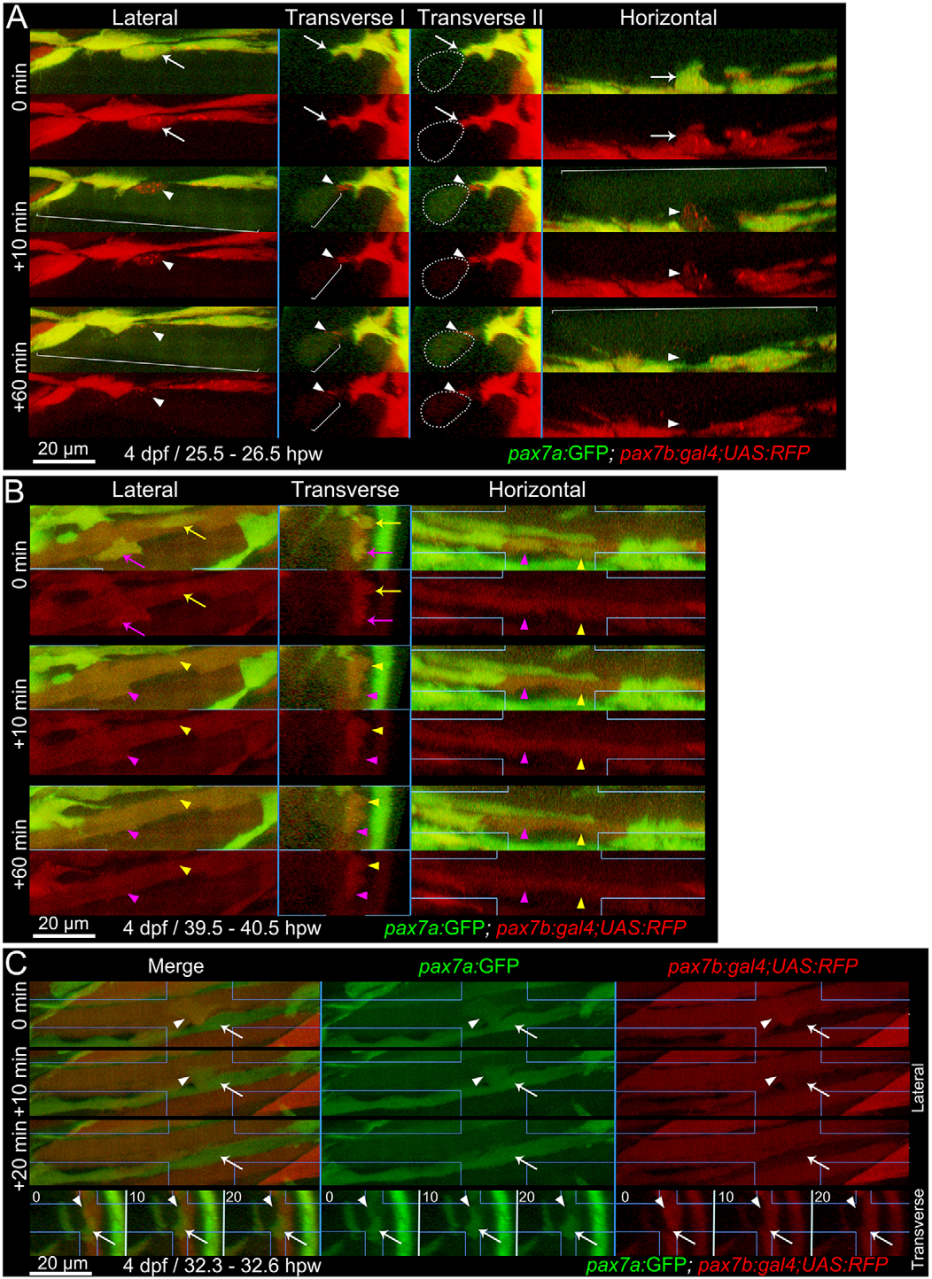
**Fusion of *pax7a*- and *pax7b*-reporter cells to existing myotubes during wound repair.** Extended orthogonal projection views of an epaxial somite wound in a *pax7a:GFP;pax7b:gal4;UAS:RFP* 4 dpf larva showing individual *pax7a:GFP;pax7b:RFP* dual-labelled (yellow) MPCs fusing to existing unlabelled (A) or RFP^+^ (B) fibres from Movies 2 and 3. The whole image was non-linearly enhanced and brightness corrected to compensate for bleaching and facilitate tracking of individual cells, as described in Materials and Methods. (A) At 25.5 hpw, prior to fusion, an MPC had uniform cytoplasmic GFP and diffuse cytoplasmic and vesicular RFP (arrow). 10 min later, cytoplasmic GFP and RFP have now filled the whole cytoplasm of a large adjacent previously unlabelled fibre (brackets), whereas the vesicular RFP remains localised (arrowhead) and integrates into the fibre in the succeeding 50 min (see Movie 2, blue MPC). Transverse II shows the same view as Transvere I, but with the fusing fibre marked (dots). (B) At 39.5 hpw, two MPCs (magenta and yellow arrows), fuse simultaneously to the same large adjacent myotube (arrowheads; Movie 2, magenta and yellow MPCs). (C) At 32.3 hpw, a dual-labelled MPC (arrowhead; Movie 3, white MPC) that originated from the anterior somite border, divided and then migrated along a recently formed GFP^+^ nascent myofibre (arrow). 10 min later the MPC has fused into the nascent fibre, as shown by RFP loss from the MPC and increase in the fibre. The fused cell remains distinct at 10 min, but merges into the nascent fibre by 20 min. Process shown in merge and single colour lateral (dorsal up, anterior left) and transverse (dorsal up, medial left) views. Blue lines indicate range of extended orthogonal projection views.

To understand whether disappearance of dual-labelled cells reflected loss of markers, cell death, migration or fusion, we performed continuous time-lapse analysis between 1 and 2 dpw. Dual-labelled MPCs were highly dynamic and frequently fused to pre-existing fibres (Fig. 6; Movie 2). Upon fusion, GFP and some RFP immediately filled the host fibre, as predicted from the rapid cytoplasmic GFP diffusion in fibres (Bajanca et al., 2015). However, the fortuitous localisation of a portion of the RFP in punctate structures within some MPCs allowed us to track the location of the fusing MPC and its integration over an hour into the fibre outline (Fig. 6A,B). *pax7a:GFP*-only MPCs also moved over and between pre-existing fibres, frequently extending and retracting processes and dividing. In continuous 3D time-lapse, two *pax7a:GFP*-only MPCs can be seen to extend processes to each somite border and form a nascent fibre (Movie 3). Simultaneously, a *pax7a:GFP;pax7b:RFP* dual-labelled cell leaves the VMZ, divides and one daughter then fuses to the nascent green fibre, which gradually accumulates RFP. Thus, migratory dual-labelled MPCs fuse into nascent fibres (Fig. 6C; Movie 3). Fibre initiation by dual-labelled MPCs was not observed. We conclude that, within wounds, dual-labelled MPCs are rapidly dividing, differentiating and fusing to fibres, consistent with the abundant labelling of fibres by *pax7b:RFP* (and weak *pax7a:GFP*), whereas *pax7a*-only cells behave differently, aligning less in wounds and initiating fibre formation.

### Ablation of Pax7b-expressing cells reveals pax7a:GFP cell behaviour

To examine the behaviour of *pax7a:GFP*-only cells in the absence of dual-labelled cells, we ablated *pax7b*-expressing cells using the nitroreductase/metronidazole (NTR/MTZ) system (Curado et al., 2007). Treatment of *pax7a:GFP;gSAIzGFFD164A;UAS-E1b:NTR-mCherry* larvae with MTZ overnight eliminated most mCherry-labelled MPCs and led to numerous phagocytes containing red debris in the ventral regions (Fig. S10). MTZ had no effect on larvae in the absence of *NTR-mCherry*. The *UAS:NTR-mCherry* transgene marked slightly fewer cells than the *UAS:RFP* or *UAS:GFP* reporters, explaining why not every *pax7b*-expressing cell was eliminated (Fig. 5D; Fig. S10). In *pax7a:GFP;pax7b:NTR-mCherry* larvae, *pax7a:GFP* cells were diminished by MTZ treatment, consistent with the presence of GFP in many *pax7b:NTR-mCherry* cells, but substantial numbers of *pax7a:GFP*-only cells remained at 3 dpf. Thus, MTZ efficiently and selectively eliminates most *pax7b*-expressing cells.

**Fig. 7.**
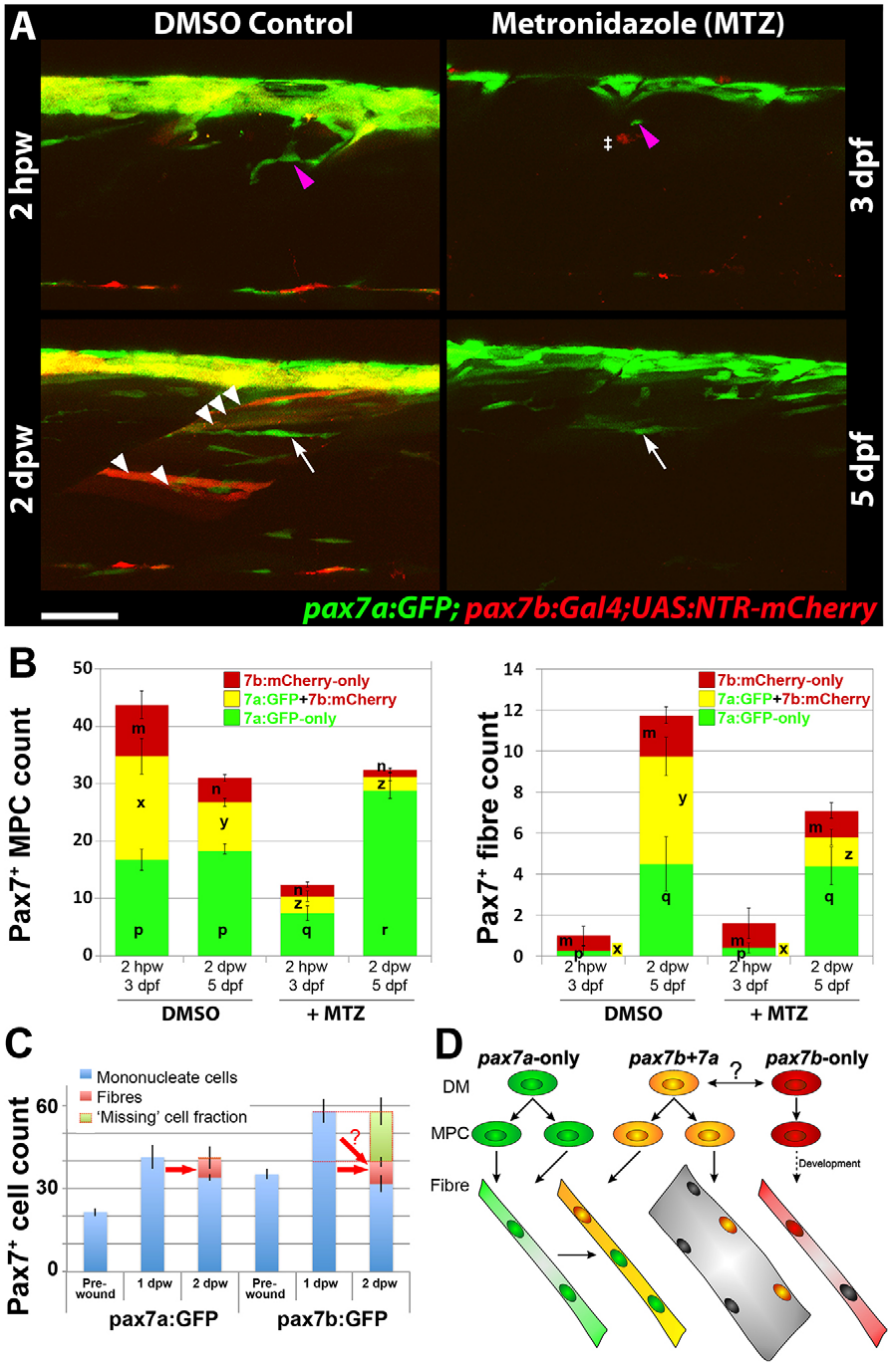
**Ablation of *pax7b*-expressing cells diminishes marked fibres.** *pax7a:GFP;pax7b:NTR-mCherry* larvae were treated overnight with MTZ or DMSO vehicle, then wounded and imaged by confocal 3D scanning at the indicated times. (A) Representative time-lapse images of wounded epaxial somites, showing MPC (magenta arrowheads) invasion of wound at 2 hpw, followed by formation of nascent fibres at 2 dpw (white arrows). Numerous large dual-labelled fibres are observed in control (white arrowheads). Anterior to left, dorsal to top. Scale bar: 50 µm. (B) Counts of numbers (mean±s.e.m.) of red, green and dual-labelled MPCs and fibres in single wounded epaxial somites of control and MTZ-treated larvae. Counts omitted fast-moving phagocyte (‡ in A) and transient fibre labelling, which appeared rapidly only after wounding in MTZ-treated NTR-mCherry larvae and reflect the abundant release of mCherry from dying cells and uptake into injured fibres or phagocytes. Counts of each labelled cell type were compared between DMSO- and MTZ-treated larvae at 2 hpw and 2 dpw by two way ANOVA (with Bonferroni post hoc test, *n*=4 DMSO and 5 MTZ; for complete dataset and statistical analyses see Table S1). Within each cell type, distinct letters on columns (m,n,p,q,r,x,y,z) show groups that differed statistically at *P*<0.05. (C) Quantification (mean±s.e.m.) of mononucleate cells in epaxial wounded somites of *pax7a:GFP* or *pax7b:GFP* larvae reveals an increase at 1 dpw, followed by a decrease at 2 dpw, accompanied by formation of labelled fibres. Note the similar number of labelled fibres in each line (red arrows), and the apparent substantial reduction of labelled cells (‘missing’ fraction) only in the *pax7b*:*GFP* reporter line. (D) Modified founder cell model illustrating the known and hypothesised (?) behaviour of the *pax7a:GFP*-only MPC population (green), which contribute to wound repair and form nascent fibres, and the *pax7b* MPC population (yellow), which is more abundant, fuses to pre-existing (damaged?) fibres in the region of the wound and contributes to nascent fibre growth. We hypothesise that *pax7a*-only cells are founders initiating new fibre formation, whereas *pax7b* cells are FCMs contributing to fibre growth both in wounding and normal development. *pax7b:RFP*-only MPCs (red) form fibres early in development, accumulate at HZM and might act as a stem cell.

When untreated larvae were wounded, numerous *NTR-mCherry*-labelled fibres arose within the wound at 2 dpw (Fig. 7A,B). In contrast, when MTZ-treated larvae were wounded, few *pax7b:NTR-mCherry*-labelled fibres arose within the wound, consistent with the ablation of most *pax7b:NTR-mCherry* MPCs (Fig. 7A,B; Table S1). Nevertheless, remaining *pax7a:GFP*-only cells accumulated in the wound (Fig. 7A). Strikingly, however, no recovery of *NTR-mCherry*-labelled cells was observed until at least 3 dpw, demonstrating that *pax7a:GFP*-only cells did not give rise to *pax7b*-expressing cells (Fig. 7A,B). Within wounds, *pax7a:GFP*-only cells formed thin nascent fibres expressing GFP, both in MTZ-treated and untreated larvae (Fig. 7A,B; Table S1). At 1 and 2 dpw, *pax7a:GFP* cells were more numerous in wounded somites in the absence of *pax7b:NTR-mCherry* cells than in their presence, suggesting rapid proliferation of remaining *pax7a:GFP* cells (Fig. 7B; Table S1 and data not shown). Nevertheless, the extra *pax7a:GFP*-only MPCs did not give rise to additional GFP-only fibres compared with non-ablated injured controls, at least prior to 3 dpw, the latest time point examined (Fig. 7B; Table S1). In contrast, MTZ greatly reduced formation of new dual-labelled fibres, consistent with the reduction in *pax7b*-expressing MPCs (Fig. 7B; Table S1). Surprisingly, despite the absence of *pax7b:NTR-mCherry*-labelled fibres and lack of compensating increase in *pax7a:GFP* fibres, the gross morphology of wounds in MTZ-treated fish did not appear worse than that of untreated wounded larvae at 2 dpw (data not shown). These results show that *pax7a:GFP*-only cells do not convert to *pax7b*-expressing cells within wounds and do not substitute for the depletion of the latter cells by enhanced differentiation.

### Fusogenicity of *pax7b*-expressing cells

As already described (Fig. 5C,E, Fig. 6), *pax7b:GFP* MPCs align more often in rows than *pax7a:GFP*-only cells, suggesting different potential fusogenic behaviour. Several additional differences between *pax7a:GFP*-only and the *pax7b:GFP* MPCs support this view. Firstly, whereas both populations of precursor cells had increased in number to a similar extent at 1 dpw, by 2 dpw *pax7b:GFP* cell numbers had returned to the unwounded value, but bright *pax7a:GFP* cell numbers remained elevated (Fig. 7C), indicating a greater tendency to differentiate and fuse in *pax7b:GFP* MPCs. Secondly, the decrease in *pax7a:GFP* MPC numbers between 1 and 2 dpw was, within counting error, similar to the number of new *pax7a:GFP* fibres formed; there were no ‘missing’ MPCs. In contrast, the decrease in *pax7b:GFP* MPC numbers was greater than the increase in the number of *pax7b:GFP* fibres at 2 dpw; the difference we termed ‘missing’ MPCs (Fig. 7C). As neither apoptosis nor gradual loss of label of *pax7b:GFP* cells were observed (e.g. Movie 2), the apparently ‘missing’ fraction of *pax7b:GFP* MPCs might have fused with one another to form multinucleate fibres. Lastly, when a GFP^+^ MPC fused to a pre-existing fibre, the GFP signal immediately became much weaker. This argues that large fibres with bright fluorescence derive from fusion of multiple marked MPCs; such bright fibres are rare in *pax7a:GFP* but more common *pax7b:GFP* fish (Fig. 4B). Taken together, these data suggest that *pax7a:GFP* cells contribute less to fibre repair in wounds than do *pax7b:GFP* cells.

## DISCUSSION

Our findings on muscle wound repair lead to three major conclusions. First, that the process and timing of muscle repair in larval zebrafish has great similarities to that in adult mammalian muscle. Second, that duplicated *pax7a* and *pax7b* genes in zebrafish provide molecular markers of MPC cell lineage heterogeneity. Third, that each population of MPCs had specific behaviours in wound repair that suggest a modified founder cell/fusion competent myoblast model operates in vertebrates.

### Visualisation and conservation of muscle wound repair in vertebrates

Our characterisation of the time course of muscle regeneration in zebrafish larvae reveals remarkable similarities with that in adult fish and mammals. Extending previous studies of larval zebrafish muscle repair (Seger et al., 2011; Knappe et al., 2015), we show that epidermal wounds close within hours and leukocytes marked by transgene reporters of both neutrophil (*mpx*) and macrophage (*lyz*) genes infiltrate the muscle abundantly but transiently for around two days, a time course comparable with that observed in other model organisms and human (Ciciliot and Schiaffino, 2010). Pax7-marked MPCs are triggered to enter the wound, divide and undergo terminal differentiation involving Myogenin upregulation, and regenerate muscle fibres. In small wounds, new muscle fibres form from 1 dpw and show significant repair by 2 dpw, which is somewhat faster than reported in mammalian models (Ciciliot and Schiaffino, 2010). However, in larger wounds, comparable with those analysed in mammalian systems, we observe slower repair, with fibres regenerated progressively from the wound edge and taking around a week, a time course comparable with that in the large wounds generally studied in other species (Ciciliot and Schiaffino, 2010). As satellite cell-based muscle repair is a synapomorphy of vertebrates (Chen et al., 2006; Hollway et al., 2007; Zhang and Anderson, 2014), our findings validate use of zebrafish to study mechanisms of muscle regeneration.

Hitherto, direct visualisation of MPC fusion during muscle repair has not been reported. Our imaging of MPC fusion directly to both pre-existing and nascent fibres in wounded regions shows that the cell biology of fusion and regeneration in a 3D mesenchymal tissue *in vivo* is accessible in the zebrafish. Fusion has been imaged in detail in *Drosophila* muscle development (Kim et al., 2015; Richardson et al., 2008); from our initial analysis, the vertebrate process appears similar. We find that MPCs fuse laterally to fibres at any point along their length, and the process is rapid, occurring in a few minutes and as little as 3 h after the final MPC mitosis. Both slow and fast fibres regenerate, but our analysis focused on the multinucleate fast fibres; how MPCs regenerate slow fibres remains to be determined.

### Pax7 genes as molecular markers of MPC cell lineage heterogeneity

Our studies reveal several zebrafish MPC populations, based on differential expression of the *pax7a* and *pax7b* genes in distinct somitic locations, lack of interconversion between MPC sub-populations and different cell behaviours in response to wounding. Endogenous *pax7a* and *pax7b* genes are differentially expressed in embryonic MPCs, with *pax7b* expressed in early dermomyotome precursors in the anterior somite border and *pax7a* expressed later (Hammond et al., 2007; Minchin and Hughes, 2008). Genetic marking confirmed the differential expression and revealed that MPCs expressing *pax7a:GFP*-only, *pax7b:reporter*-only or both are found in larvae. In the absence of antibodies specific to each Pax7 protein it is unclear whether the reporters reflect endogenous protein accumulation. Nevertheless, the markers can be used to track the fate of each MPC type.

Within wounds, *pax7a:GFP*-only cells participate in repair, forming nascent fibres, but their GFP rapidly diminishes in regenerated fibres, apparently by dilution as newly formed fibres enlarge. In contrast, *pax7b*-reporters (we used several) persist in regenerated fibres. *pax7b*-only cells are rare in wounds; most *pax7b:RFP* MPCs also contain detectable *pax7a:GFP*. These dual-labelled cells frequently fuse to fibres. As Pax7 immunoreactivity was not observed in muscle fibres, this persistence of *pax7b:GFP*/*RFP* is best explained by perdurance of GFP/RFP, perhaps from more abundant or ongoing MPC fusion. In summary, within wounds, *pax7b* expression distinguishes *pax7a*-only and dual-labelled MPC populations.

Each MPC population is stable within wounds. Interconversion was not observed in time-lapse studies. Moreover, even when *pax7b*-expressing cells are ablated, *pax7a*-only MPCs do not regenerate dual-labelled MPCs. The two populations express *pax7a:GFP* differently. In general, *pax7a:GFP*-only MPCs tend to have more GFP than dual-labelled MPCs. Consequently, without sensitive equipment the *pax7a:GFP*-only cells are preferentially detected in the GFP channel and appear to form mostly small nascent fibres. Although all MPCs in wounds express some level of p*ax7a:GFP*, *pax7b:GFP* marks more, and larger, regenerated fibres. This suggests that *pax7b:GFP* perdures better than *pax7a:GFP* and/or that more *pax7b:GFP* cells fuse. Taken together, the data argue that the two populations of MPCs represent distinct cell lineages that respond differently to wounding.

In aggregate, the two MPC populations explain the results observed with Pax7 protein. Confirming previous studies (Knappe et al., 2015; Seger et al., 2011), we show that Pax7^+^ cells are more abundant than bright *pax7a:GFP*-MPCs, accumulate and proliferate in muscle wounds and express markers of myogenic progression, such as Myf5 and Myogenin. Our quantitative analysis showed that around half of all somitic Pax7^+^ cells in wounded somites co-express Myogenin, a marker of terminal differentiation. As ∼40% Pax7^+^ were in S-phase at 1 dpw, it seems that most non-differentiating Pax7^+^ cells must be proliferating rapidly, explaining the increase in Pax7^+^ cells and recovery of cell number in wounds. Congruently, cells marked by either *pax7a* or *pax7b* reporters often divided in wounds. Even in large wounds, when repair is nearing completion, both MPC populations recover at the somite borders, as expected of muscle stem cells.

When *pax7b:NTR-mCherry*-marked MPCs are ablated they do not recover, nor do *pax7a:GFP*-marked MPCs contribute more GFP to repaired fibres in wounds. Strikingly, however, recovery of overall muscle morphology in these small wounds was not grossly defective. Whether a regeneration defect in *pax7b*-reporter MPC-ablated muscle causes a persistent change in myotome cellularity will require further quantitative analyses.

The range of MPC migration is a key factor affecting muscle growth, regeneration and the effectiveness of gene and cell therapies (Bentzinger et al., 2014; Hughes and Blau, 1990; Negroni et al., 2015; Partridge et al., 1989). Our data show that at the time of wounding (∼3 dpf), there are few Pax7^+^ cells deep within the body of the myotome, but within a few hours after injury *pax7a-* and *pax7b*-reporter-labelled cells migrate towards and deep into the wounded region. Moreover, whereas Pax7^+^ cell numbers in the central region of the somite regained or exceeded control levels within 1 dpw, there was a striking delay in the recovery of Pax7^+^ cells on the VMZ, consistent with migration of a proportion of the cells into the body of the regenerating somite from the borders. Although new fibre formation during larval growth occurs in specific somitic locations (Barresi et al., 2001; Johnston et al., 2009), we observed efficient regeneration irrespective of wound location within the epaxial somite, demonstrating that resident MPCs can rapidly reach most somite regions. The wounded zebrafish somite might provide a suitable *in vivo* screening system for factors regulating MPC migration.

In unwounded somites, *pax7a*-only cells accumulate at VMZ and the dorsal and ventral myotome edges. After wound regeneration, we observed particularly numerous *pax7a:GFP* cells at the posterior border of regenerated somites, a location suggested to contain abundant myogenic precursors in various fish species (Marschallinger et al., 2009). Conversely, *pax7b-*reporter cells are less abundant in these locations but are more numerous at the HZM and in cells scattered over the lateral myotome surface, the location of the zebrafish dermomyotome (Devoto et al., 2006). In amniotes, cells derived from the central and border dermomyotome regions behave differently, but both express Pax7 (Ben-Yair and Kalcheim, 2005; Buckingham and Relaix, 2007; Gros et al., 2005; Schienda et al., 2006). The expression of *pax7a:GFP* alone in certain neural tube cells, also suggests sub-functionalisation of each gene during teleost evolution. To conclude, we hypothesise that subpopulations of Pax7^+^ MPCs corresponding to those we have revealed in zebrafish also exist in amniotes (Fig. 7D), and might have retained evolutionarily conserved functional roles in both muscle growth and wound repair.

### A modified founder cell/fusion competent cell model in vertebrates

What roles do the two populations of MPCs play? In *Drosophila*, genetically defined individual founder cells initiate each fibre, which then grows by fusion with numerous fusion-competent myoblasts (FCMs) (Atreya and Fernandes, 2008; Dutta et al., 2004; Rushton et al., 1995). Our data provide evidence for a modified founder cell/FCM system in zebrafish muscle regeneration. Founder cells have not been described in vertebrates. However, clones of vertebrate embryonic myoblasts only fuse to form small nascent fibres with few nuclei (Miller and Stockdale, 1986). Later-arising clones generate large multinucleate myotubes (Cossu et al., 1988; Miller and Stockdale, 1986). Moreover, the initial fusion events that form mammalian myotubes and the subsequent growth of myotubes by fusion are differentially regulated (Horsley et al., 2001, 2003). We find that MPC expressing *pax7a:GFP*-only migrate early to wounds and differentiate to mark thin, presumably nascent, myotubes from 1 dpw onwards, even when *pax7b*-expressing cells are ablated. Such cells can have one or a few nuclei. In contrast, *pax7b*-expressing MPCs also migrate to wounds early, but rapidly contribute to both small and large fibres by 1 dpw, frequently fuse to pre-existing fibres and also align in rows reminiscent of fusing myoblasts. Most convincingly, our time-lapse Movie 3 captures the entire process of individual *pax7a*-only MPCs initiating a fibre and then a *pax7b*-expressing MPC fusing into the nascent fibre. Moreover, *pax7b* reporters persist in marking larger fibres at later stages of repair. The greater reduction in *pax7b:GFP* cell numbers between 1 and 2 dpw strongly suggests that many of them fuse with one another. All these data argue that, during regeneration, the less-abundant *pax7a*-only MPCs initiate nascent fibre formation, whereas more numerous *pax7b* MPCs contribute to the growth of nascent fibres and to the repair of damaged pre-existing fibres (Fig. 7D). Our vertebrate ‘modified founder cell hypothesis’ asserts that (1) a unique lineage of founder cells initiate formation of a fibre with or without fusion to one another, (2) the nascent fibre then grows by addition of myoblasts from a second distinct lineage of MPCs. In zebrafish larval regeneration, *pax7a*-only cells, and *pax7b*-expressing cells (most of which also express *pax7a*) behave like founders and FCMs, respectively.

In amniotes, MPC heterogeneity has long been thought to underpin generation of distinct kinds of muscle cells during development (Cossu et al., 1983; Miller et al., 1985; Rutz and Hauschka, 1982; White et al., 1975). An advantage of the duplication of the *pax7* gene in the zebrafish is that it reveals the distinct behaviour of *pax7a:GFP*-only and *pax7b:GFP* cells, suggesting they might represent distinct cell lineages with particular roles in myogenesis. Complex somitic myogenesis with distinct waves of fibre formation and a dermomyotome-like stem cell compartment arose prior to the divergence of teleost and amniote ancestors (Devoto et al., 2006). We hypothesise, therefore, that several Pax7^+^ lineages might have existed in the common ancestor and could persist in extant amniotes. However, as amniotes only have a single (unduplicated) *Pax7* gene, distinction of two MPC sub-populations comparable to those marked with *pax7a:GFP*-only and those expressing *pax7b* (with or without *pax7a*) in fish, might have gone unnoticed hitherto. Given that the earliest reported muscle phenotype of *Pax7* single knockout mice is loss of quiescent satellite cells postnatally (Relaix et al., 2004, 2005; von Maltzahn et al., 2013), it is unclear whether either fish *Pax7* gene is essential for myogenesis in zebrafish at the stages we examined. Several other MPC populations have been suggested to exist in zebrafish (Seger et al., 2011; Siegel et al., 2013), but their relationship to *pax7a*- and *pax7b*-marked cells is currently unclear. If the cell lineages we have discovered are indeed the vertebrate equivalent of founder cells and FCMs, we expect such lineages to persist throughout life in all vertebrates. By analogy with *Drosophila*, one might also expect that additional diversity in the founder cell population underlies muscle identity and/or fibre type diversity.

## MATERIALS AND METHODS

### Zebrafish lines, maintenance and manipulation

Zebrafish (*Danio rerio*) embryos were kept and staged by standard methods (Kimmel et al., 1995; Westerfield, 2000). *Tg(-2.2mylz2:gfp)^i135^* (von Hofsten et al., 2008), *Tg(9.7kb smyhc1:gfp)^i104^* (Elworthy et al., 2008), *Tg(pax7a:GFP)* (Mahalwar et al., 2014), *pax7b^gSAIzGFFD164A^;5xUAS:EGFP^1A^* or *5xUAS:RFP^1A^* (Asakawa et al., 2008), *Tg(UAS-E1b:NTR-mCherry)^c264^* (Davison et al., 2007), *Tg(h2afva:H2AFVA-GFP)^kca66^* (Pauls et al., 2001), *Tg(Ola.Actb:Hsa.HRAS-EGFP)^vu119^* (Cooper et al., 2005) were maintained on King's wild-type or AB background. *Tg(lyz:EGFP)^nz117^* (Hall et al., 2007) and *Tg(mpx:GFP)*^i114^ (Renshaw et al., 2006) were on *roy;mitfa*. Care and use of experimental animals complied with all relevant institutional and national animal welfare laws, guidelines and policies.

To label Pax7b^+^ cells, we performed a transposon-mediated gene trap screen and identified the *pax7b* trap line in which the Gal4FF is integrated in the fourth intron of the *pax7b* gene: *pax7b^gSAIzGFFD164A^;5xUAS:EGFP* (Asakawa et al., 2008). Kaede injections were as described (Minchin et al., 2013). For wounding, dechorionated larvae were embedded on their side in 1% low melting point agarose (LMA) in embryo medium (EM) containing 160 mg/l MS222 anaesthetic (Westerfield, 2000) and damaged by insertion of a fine glass needle with a tip diameter of 15-20 µm at 45° into one or more epaxial somites at the end of the yolk extension using a Narashige M153 micromanipulator. The underlying notochord and neural tube were avoided, as accidental damage of these tissues can trigger death. Controls were mounted but uninjured larvae from the same lay. After wounding, each fish was released from LMA, kept separately and daily re-embedded for imaging. Penicillin (50 units/ml) and streptomycin (50 µg/ml) and 0.2 mM phenylthiourea were sometimes added to reduce infection and enhance imaging, but did not observably affect wound behaviour. Cells in S-phase were labelled for 3 h in 4 mM or 3 d in 50 µM 5-ethynyl-2′-deoxyuridine (EdU) and detected with Click-iT (Invitrogen). To ablate cells *pax7b^gSAIzGFFD164A^;UAS:NTR-mCherry* larvae were treated overnight with 2.5-10 µM metronidazole at 2.5 dpf and wounded at 3.5 dpf.

### Immunodetection

Larvae were fixed with 2% paraformaldehyde in PBS for 25 min to overnight depending on the stage. Immunodetection for slow myosin heavy chain (MyHC) (1:5, F59; Devoto et al., 1996), general MyHC (1:10, A4.1025; Blagden et al., 1997), Pax7 (1:5; DSHB; Kawakami et al., 1997), Myogenin (1:50, sc-576, Santa Cruz) and GFP (1:500, TP-401, Torrey Pines or 1:500, G1546, Sigma) was performed in PBS with 0.5-1% Triton X-100 (PBT) for between overnight and 5 days at 4°C on a rotary shaker, depending on larval age and antigen (Hinits and Hughes, 2007) followed by Alexa Fluor 488 or 555 secondary antibodies (1:1000; A21121 and A21428, respectively; Invitrogen). at least overnight (±Hoechst 33342) at 4°C. After EdU detection in Fig. S4, samples were blocked in 5% BSA, 3% normal goat serum, PBT for 20 min, incubated using Alexa Fluor 488-conjugated anti-GFP (1:500, A-21311, Molecular Probes) and Hoechst in block buffer (shaking at 4°C for 3-6 h), followed by 6×15 min washes in PBT. Phalloidin-Alexa Fluor 555 or phalloidin-Alexa Fluor 633 (1:1000, A34055 or A22284, Thermo Fisher) were used to-stain F-actin. Larvae were mounted on glass slides under bridged coverslips in Citifluor AF1 or Vectashield (H-1000, Vector Laboratories). *In situ* mRNA hybridisation was as described (Groves et al., 2005).

### Imaging and data analysis

Time-lapse fluorescence images were acquired on either a Zeiss LSM Exciter M1 or LSM780 with Zeiss 20X/1.0

W PlanApochromat or LD C-Apochromat 40x/1.1 W objectives using Zen software. Larvae were mounted in 1% low melting point agarose (LMA) in EM containing 160 mg/l MS222 anaesthetic (Westerfield, 2000) and antibiotics (Sigma, P0781 used at 1:100) in a 60 mm Petri dish, flooded with EM (upright LSM) or 20 mm glass-bottom dish (inverted LSM). Where scan intervals allowed, larvae were removed from LMA and MS222 between time points and returned to a 28.5°C incubator. Image analysis and processing was done with Volocity 6.3 (PerkinElmer), Imaris 8.2 (Bitplane), Photoshop CS5 (Adobe) and Fiji/ImageJ (NIH). In movies, to compensate for bleaching and facilitate cell tracking of individual cells, the whole image stacks were brightened and non-linearly enhanced by altering gamma in Imaris, so as to produce comparable brightness within the images at each time point. No quantitation was done on non-linearly manipulated data. For some live imaging experiments, embryos were injected at the 1-cell stage with ∼150 pg RNA encoding plasma membrane-targeted mCherry (Shaner et al., 2004) yielding ubiquitously red cell membranes. Nuclear counts were means for all wounded or a similar number of adjacent unwounded somites from each animal. Half the cells on both VMZs and HZM were attributed to an epaxial somite. GFP^+^ cell numbers were analysed in epaxial somite before and after focal needle wounds in *pax7a:GFP;pfe/pfe, pax7b:GFP* or *pax7a:GFP;pax7b:red reporter* larvae from confocal image stacks repeatedly collected at 1.5-24 h intervals. Mononucleate cells were defined as a volume of uniform signal with little or no contact with neighbours, or with a distinguishable level of GFP. Cell alignment, defined as the projected long axes of two cells reciprocally entering the fluorescent region of the neighbour cell within the same somite, was assessed by eye, unblinded (as *pax7a* and *pax7b* reporters are readily distinguished) in Volocity 3D stacks using ‘ortho’ view. New fibres were defined as elongated cells spanning the length of the somite with uniform reporter intensity that were not present at 3 hpw. Pre-existing *pax7b:GFP*-marked fibres were not counted. ‘Missing’ cells=MPCs at 1 dpw−(MPCs at 2 dpw+Fibres at 2 dpw). Graphs show means and standard error of the mean (means±s.e.m.) for the number of individual embryos shown. Statistical analysis was done with Microsoft Excel (Student's *t*-test, after *F*-test for un/equal variance), AnalystSoft StatPlus v.5 or IBM SPSS (ANOVA with Scheffé/Tukey/Bonferroni post-hoc and non-parametric tests where appropriate).
